# Efficient Unrestricted Identity-Based Aggregate Signature Scheme

**DOI:** 10.1371/journal.pone.0110100

**Published:** 2014-10-20

**Authors:** Yumin Yuan, Qian Zhan, Hua Huang

**Affiliations:** 1 School of Applied Mathematics, Xiamen University of Technology, Xiamen, China; 2 University of Science and Technology Beijing, Beijing, China; 3 University of Xiamen, Xiamen, China; University of Catania, Italy

## Abstract

An aggregate signature scheme allows anyone to compress multiple individual signatures from various users into a single compact signature. The main objective of such a scheme is to reduce the costs on storage, communication and computation. However, among existing aggregate signature schemes in the identity-based setting, some of them fail to achieve constant-length aggregate signature or require a large amount of pairing operations which grows linearly with the number of signers, while others have some limitations on the aggregated signatures. The main challenge in building efficient aggregate signature scheme is to compress signatures into a compact, constant-length signature without any restriction. To address the above drawbacks, by using the bilinear pairings, we propose an efficient unrestricted identity-based aggregate signature. Our scheme achieves both full aggregation and constant pairing computation. We prove that our scheme has existential unforgeability under the computational Diffie-Hellman assumption.

## Introduction

An aggregate signature [1] is a useful primitive that allows anyone to compress *n* individual signatures, say 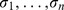 where 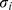 is a signature from user with identity 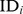 on message 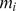 for 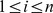, into a single (shorter) signature even if these signatures are on the same message or are produced by the same signer. The main goal in the design of such protocols is to reduce the costs on storage, communication and computation. Informally, the length of the aggregate signature should be constant, independent of the number of messages and signers. The resulting signature can convince a verifier that the user 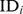 indeed signed the corresponding message 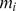 for all 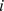: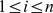. This primitive is useful in many real-world applications (which involve multiple signatures on multiple messages generated by multiple users) especially in environments with low-band-width communication, low-storage and low computability. Typical applications for such schemes are Wireless sensor networks (WSNs) since WSNs are resource constraint: limited power supply, bandwidth for communication, memory space [2]. For example, in an environment monitoring network, the sensors record measurements from the environment, sign its data and send them to a monitoring center. The center aggregates these data and the signatures to save storage [3]. Aggregate signature scheme can also be applied to vehicular communications [4], many-to-one authentication [5], electronic transactions [6] and cloud computing [7] to enhance the efficiency of verification and reduce the communication over-head.

Boneh, Gentry, Lynn and Shacham [1] first defined of aggregate signature and presented a concrete aggregate signature which was constructed under traditional public key cryptography (PKC). In traditional PKC, a digital signature provides the authenticity of a signed message with respect to a public key, while the authenticity of the public key with respect to a signer is contained in a certificate provided by a certificate authority (CA). Whenever a verifier wants to verify a signature, he has first to verify the corresponding certificate. Therefore, aggregate signature working under traditional PKC requires heavy management, communication and computation cost to achieve authenticity of all signers’ public keys, making the scheme both space and time inefficient, especially when the number of signers is large. To reduce this burden, Shamir [8] proposed the concept of identity-based public key cryptography (IB-PKC). The IB-PKC requires a trusted third party, typically called a “Private Key Generator” (PKG) which serves a similar role to the CA in a PKC system, to generate system parameters and user’s private key. In an identity-based cryptosystem, only the PKG has a traditional public key, and the public key of each user is derived directly from his identity information, such as his email address. The direct derivation of users’ public keys in these infrastructures eliminates the need for the certificate and some of the problem associated with them. In an identity-based signature (IBS) scheme, to generate valid signatures of a signer with the identity ID, one needs to know the private key of ID, while verifier can directly use the signer’s identity ID and the PKG’s public key to verify signatures. This advantage of identity-based aggregate signature (IBAS) becomes more compelling when we consider multiple signers. In this setting, when all signers have their secret keys issued by the same private key generator (PKG), the verifier needs only one traditional public key (of the PKG) to verify multiple identity-based signatures on multiple messages.

To shorten the length of signatures and to avoid the authentication of the public keys, Cheon et al. [9] presented the first identity-based aggregate signature (IBAS) scheme. To date several IBAS schemes have been proposed [9]–[20]. However, some of them have additional restrictions conditions on aggregation step. The schemes [10], [11] do not support simultaneous aggregation, which only allow each signer to aggregate his signature to a previously aggregated signature in turn. The scheme [12] requires that all signers participating in aggregation have to agree upon a common random string which was never used by any of the signers. Secure use of the scheme [12] is restricted to the aggregation of signatures from distinct signers. The scheme [13] requires interactive communication between signers to generate an aggregate signature, and hence increases the communication complexity.

Among existing unrestricted aggregate signature schemes (which enable any user to freely aggregate multiple signatures) in the identity-based setting [9], [14]–[20], all but one of them [9], [14]–[19] are able to achieve only partial aggregation and not full aggregation, i.e., the length of the resulting aggregate signature grows with the number of aggregated individual signatures, which departs from the main goals of aggregate signatures. Obviously, such schemes are impractical for some wireless network scenarios. Only the scheme in [20] achieves constant-length aggregate signature. But this scheme requires a large number of pairing operations in which the number of pairing operations in the aggregate signature verification algorithm is proportional to the number of aggregated individual signatures.

In this paper, we construct an efficient IBAS scheme without any restriction. The proposed protocol is based on bilinear pairings. The new scheme simultaneously achieves constant-length aggregate signature and constant pairing operations during signature verification, and is shown to be existentially unforgeable against adaptive chosen message attacks under the computational Diffie-Hellman assumption in the random oracle model.

## Preliminaries

In this section, we review the basic concept of bilinear pairings and the complexity assumption on which our scheme relies.

### 2.1 Bilinear pairings

Let 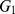 be a cyclic additive group of prime order *q* and 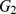 be a cyclic multiplicative group of the same order. A map 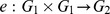 is called a bilinear pairing if it satisfies the following properties:

1. Bilinear: 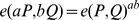 for all 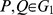 and all 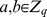.
2. Non-degeneracy: There exist 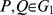 such that 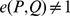.
3. Computable: There is an efficient algorithm to compute 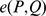 for any 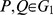.

### 2.2 Related complexity assumption

#### Definition 1

A function : *N* →*R* is said to be negligible if, for every positive polynomial *poly* (*·*) there exists an integer 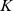
> 0 such that for all 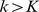 it holds.



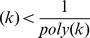



Otherwise, we call  non-negligible.

#### Definition 2

Let *G* be a group of prime order 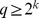 where *k* is a security parameter. Computational Diffie-Hellman (CDH) Problem is that given three elements 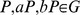 for unknown randomly chosen 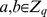, compute 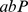.

Let 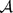 be a probabilistic polynomial-time algorithm. The advantage of 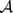 in solving the CDH problem in group 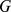 is defined to be.
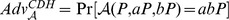
where the probability is taken over the uniformly and independently chosen instance with a given security parameter 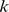 and over the random choices of 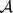.

The CDH assumption states that for every probabilistic polynomial-time algorithm 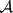, 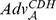 is negligible.

### Definitions and Security Models

We first review the definition and the formal security model for IBS schemes. Then we describe the definition and the formal security model for IBAS schemes.

### 3.1 Formal model of identity-based signature schemes

#### 3.1.1 Definition of identity-based signature schemes

An identity-based signature (IBS) scheme is a tuple of probabilistic polynomial-time algorithms (**Setup**, **Extract**, **Sign**, **Verify)**. The description of each algorithm is as follows.

- **Setup**. This algorithm is run by a private key generator (PKG). It takes a security parameter *k* as input and outputs a master key *msk* and a list of system parameters *params*. The system parameters will be publicly known while the master key will be known to the PKG only.
- **Extract**. This algorithm takes a user’s identity ID*_i_*, a system parameters *params* and a master key *msk* as input, and outputs the user’s private key 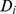. Usually, this algorithm is run by the PKG. The PKG sends 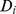 to the user ID*_i_* through a secure channel.
- **Sign**. This algorithm takes a system parameters *params*, a message *m_i_*, an identity ID*_i_* and corresponding private key 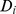 as input, and outputs an individual signature 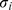 on the message *m_i_* for the user with identity ID*_i_*. This algorithm is executed by the user ID*_i_*.
- **Verify**. This algorithm takes a system parameters *params*, an identity ID*_i_*, a message *m_i_* and an individual signature 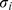 as input, and outputs 1 or 0 for valid or invalid, respectively.

#### 3.1.2 Security requirements for identity-based signature schemes

We review the usual security model of IBS [17], [21] which is an extension of the usual notion of existential unforgeability under chosen-message attacks [22]. The security model mainly captures the following two attacks:

1. Adaptive chosen message attack: It allows an adversary to ask the signer to sign any message of its choice in an adaptive way, it can adapt its queries according to previous answers;
2. Adaptive chosen identity attack: It allows the adversary to forge a signature with respect to an identity chosen by the adversary.

Finally, the adversary could not provide a new message-signature pair with non-negligible advantage. The security for an IBS scheme is defined via the following game.

#### Game I (Unforgeability of IBS)

This game is performed between a challenger 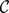 and an adversary 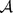 with respect to scheme (**Setup**, **Extract**, **Sign**, **Verify**), which captures the attacking scenario where a dishonest user who is allowed to have access to the signing oracle for any desired messages and identities, but he is not able to obtain victim’s private key, and wants to create a new valid signature.

#### Setup

Taking a security parameter *k* as input, the challenger 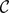 runs the Setup algorithm to obtain a master secret key *msk* and system parameters *params*. Then 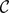 sends *params* to the adversary 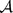, but keeps *msk* secret.

#### Queries


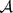
 makes a polynomially bounded number of the following queries in an adaptive manner.

- *Extraction queries*. Given an identity ID*_i_*, the challenger returns the private key 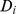 corresponding to ID*_i_*.
- *Signature queries*. Given an identity ID*_i_* and a message *m_i_*, 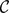 returns an individual signature 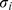 on *m_i_* with respect to ID*_i_*.

#### Forgery

Eventually, 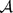 outputs an identity-based signature 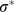 on a message 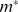 for an identity 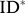. We say that 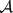 wins Game I, iff.

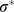
 is a valid signature on message 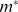 under identity 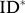.

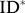
 has never been queried during the Extraction queries. And 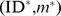 has never been queried during the Signature queries.

The advantage of 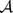 is defined as the probability that it wins in Game I.

#### Definition 3

An IBS scheme is said to satisfy the property of existential unforgeability against adaptive chosen-message attack and adaptive chosen-identity attack (EUF-IBS-CMA) if there is no probabilistic polynomial-time adversary 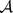 with non-negligible advantage in Game I.

### 3.2 Formal model of identity-based aggregate signature schemes

#### 3.2.1 Definition of identity-based signature aggregate signature schemes

An IBAS scheme involves a PKG, an aggregating multiset of *n* users and an aggregate signature generator. It allows the generator to compress any *n* individual signatures along with a multiset of *n* message-identity pairs, which include on the same message from the same signer, into a single signature. An IBAS scheme is a tuple (**Setup**, **Extract**, **Sign**, **Verify**, **Agg**, **AggVerify**) based on the IBS scheme (**Setup**, **Extract**, **Sign**, **Verify**) by six polynomial-time algorithms with the following functionality:

- **Setup**, **Extract**, **Sign, Verify**. These algorithms are the same as those in the IBS scheme in Section 3.1.1.
- **Agg**. This algorithm is run by an aggregate signature generator and allows the generator to compress multiple individual signatures into an aggregate signature. It takes a system parameters *params*, *n* signatures 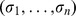 with each signature 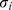 under an identity 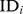 on a message 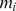 as input, and outputs an aggregate signature *σ*_Agg_ for the multiset of message-identity pairs 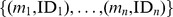.
- **AggVerify**. This algorithm takes an aggregate signature *σ*_Agg_, a multiset of *n* message-identity pairs 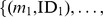 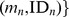 as input, and outputs 1 if the aggregate signature is valid, or 0 otherwise.

#### 3.2.2 Security requirements for identity-based aggregate signature schemes

An IBAS scheme should be secure against traditional existential forgery under adaptive chosen-message attack and adaptive chosen-identity attack. An unforgeability of IBAS is defined via the following unforgeability game which is performed between a challenger and an adversary. The adversary’s goal is the existential forgery of an aggregate signature. Informally, it should be computationally infeasible for any adversary to produce a forgery. We formalize the security model as follows.

#### Game II (Unforgeability of IBAS)

This game is performed between a challenger 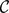 and an adversary 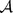 with respect to scheme (**Setup**, **Extract**, **Sign,** **Verify**, **Agg**, **AggVerify**), which captures the attacking scenario where a dishonest user who is allowed to have access to the signing oracle for any desired messages and identities, wants to create a forgery without knowing the private keys of all the signers.

#### Setup

Taking a security parameter 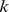 as input, the challenger 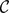 runs the Setup algorithm to obtain a master secret key *msk* and system parameters *params*. Then 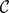 sends *params* to the adversary 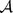, but keeps *msk* secret.

#### Queries


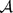
 makes a polynomially bounded number of the following queries in an adaptive manner.

- *Extraction queries*. Given an identity ID*_i_*, the challenger returns the private key 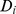 corresponding to ID*_i_*.
- *Signature queries*. Given an identity ID*_i_* and a message *m_i_*, 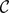 returns a signature 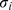.

#### Forgery

Eventually, 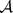 outputs a multiset of *n* message-identity pairs {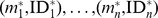} and an aggregate signature 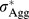. We say that 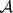 wins the game, iff.

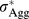
 is a valid aggregate signature on message-identity pairs 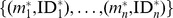, i.e., 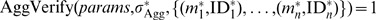.
2. At least one of the identities, without loss of generality, say 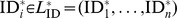 has never been queried during the Extraction queries. And 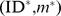 has never been queried during the Signature queries.

The advantage of 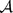 is defined as the probability that it wins in Game II.

#### Definition 4

An IBAS scheme is said to satisfy the property of existential unforgeability against adaptive chosen-message attack and an adaptive chosen-identity attack (EUF-IBAS-CMA) if there is no probabilistic polynomial-time adversary 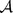 with non-negligible advantage in Game II.

## A New Identity-Based Signature Scheme

In this section, we propose a provably secure identity-based signature scheme which can be used to construct an unrestricted IBAS scheme.

### 4.1 Proposed basic identity-based signature scheme

The proposed IBS scheme consists of the following four concrete algorithms:

- **Setup.** Given a security parameter *k*, the private key generator (PKG) chooses a prime *q*, a cyclic additive group *G*_1_ and a cyclic multiplicative group *G*_2_ of prime order *q*, a random generator *P* in *G*_1_, an admissible pairing 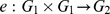, and two cryptographic hash functions 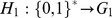 and 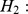 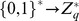. It also randomly chooses 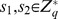, sets the master key 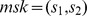, and computes *P*_1_ =  *s*_1_ *P* and 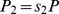. Finally, it broadcasts the system parameters, 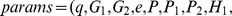 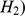.
- **Extract.** For a given identity ID*_i_*, the PKG computes 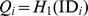 and sets this user’s private key 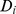 to be 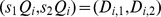.
- **Sign.** To sign a message 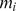 with private key 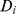, the signer with ID*_i_* chooses 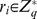 and computes 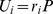, 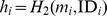, 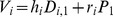 and 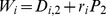. The signature on 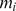 is 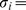 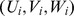.
- **Verify.** Upon receipt of an individual signature 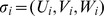, the verifier computes 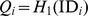 and 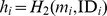, and checks 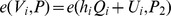 and 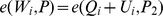. If both the equations hold, then the individual signature 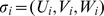 is valid.

### 4.2 Security proof of the IBS scheme

The following theorem shows that in the random oracle model, our IBS scheme is existentially unforgeable against adaptive chosen-message attack and adaptive chosen-identity attack under the assumption that CDH problem in 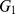 is intractable. Concretely, we show that if a probabilistic polynomial-time bounded adversary exists who can break our IBS scheme with non-negligible probability , we will be able to solve the computational Diffie-Hellman problem with non-negligible probability 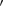, which contradicts the CDH assumption.

#### Theorem 1

In the random oracle model, if there exists a polynomial-time adversary 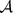 who has an advantage  in forging a signature of our IBS scheme in an attack modeled by Game I of Section 3.12 within a time at most *t*, after asking at most 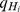 times *H_i_* (*i*  = 1, 2) queries, 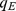 times Extraction queries and 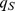 times Signature queries, then the CDH problem in 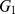 can be solved within time.
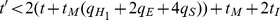
and with probability



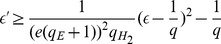
where *e* is the base of the natural logarithm, 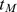 is the time of computing a scalar multiplication in 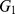, and 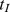 is the time of computing an inversion in 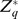.

#### Proof

Using a similar proof technique in [17], [23], [24], we are going to construct a probabilistic polynomial-time algorithm 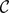 to solve the CDH problem by using the adversary 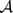 who can break our IBS scheme. Suppose that 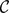 is given an random instance of the CDH problem 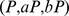 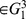 for some unknown 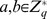. The task of 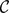 is to compute *abP*. 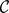 plays the role of 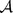
’s challenger in Game I and interacts with 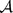 as follows:

#### Setup


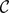
 simulates the Setup algorithm as follows:

1. Choose a random value 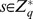
and sets *P*_1_ =  *aP*, 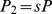, where 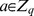 is unknown to 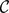.
2. Choose a cyclic group 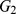 of prime order *q*, a bilinear map 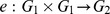.
3. Choose two hash functions *H*_1_ and *H*_2_ as random oracle.
4. Send the system parameters *params* =  (*q*, *G*_1_, *G*_2_, *e*, *P*, *P*_1_, *P*_2_, *H*_1_, *H*_2_) to 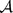.

#### Query

Proceeding adaptively, 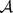 is allowed to query the random oracles *H*_1_, *H*_2_, Extraction oracle and Signature oracle in a polynomial number of times. 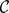 simulates these oracles for 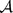 as follows:

#### *H*_1_ queries

At any time, 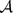 can issue an *H*_1_ query on an identity. To avoid collision and consistently respond to *H*_1_ queries, 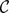 maintains a list 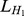 of tuples (ID, *t*, *c*, *Q*) which stores his responses to such queries. This list is initially empty. When querying the oracle *H*_1_ on ID, 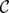 responds as follows:

1. If the query 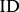 already appears on 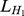 in a tuple 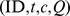,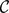 responds to 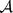 with 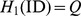.
2. Otherwise, 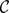 picks a random coin 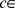 {0, 1} with Pr[*c* = 0]  = *δ*.
  - If *c* = 0, then 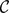 randomly chooses 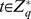 and computes *Q*  =  *t*(*bP*).
  - If *c* = 1, then 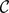 randomly chooses 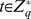 and computes *Q*  =  *tP*.

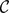
 adds the tuple (ID, *t*, *c*, *Q*) to the 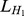 and responds to 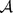 with 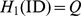.

#### *H*_2_ queries

To respond to *H*_2_ queries, 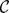 maintains a list 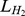 of tuples 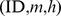, which is initially empty. When querying the oracle *H*_2_ on 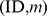, 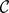 responds as follows:

1. If the query 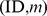 already appears on 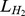 in a tuple 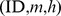, 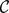 responds to 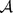 with *H*_2_(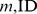)  =  *h*.
2. Otherwise, 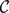 randomly chooses 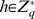, adds the tuple 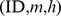 to 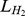 and responds to 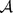 with *H*_2_(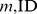) =  *h*.

#### Extraction queries

When 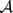 queries the private key corresponding to 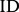, 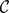 first finds the corresponding tuple (ID, *t*, *c*, *Q*) from the 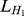.

1. If 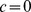, 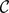 fails and aborts the simulation.
2. Otherwise, 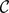 computes 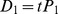 and 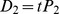, and responds to 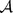 with 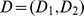.

#### Signature queries

When 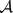 makes a Signature query on *m* for ID, 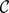 randomly chooses 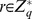 and computes 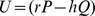, 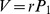. Then, 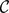 computes 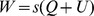 and responds to 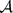 with signature *σ* = (*U*, *V*, *W*).

#### Forgery

Eventually, 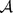 outputs a forged signature 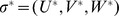 on a message 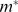 for an identity 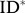. 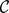 finds the corresponding tuple 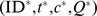 from the 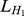. If 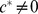, 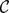 fails and aborts. Otherwise, by applying the forking lemma [25], after replaying 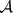 with the same random tape but different choices of oracle *H*_2_, 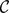 can get two valid signatures 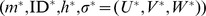 and 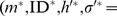 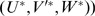 such that 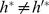. Now, since both forgeries are valid, we have
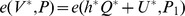


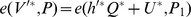



Combining the above two equations, we have
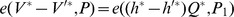



Note that 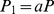 and 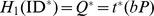 since 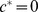. We have
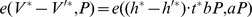


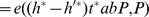



which implies
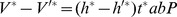



Consequently, 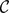 could solve the CDH by computing
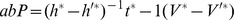



#### Probability analysis

It remains to evaluate the probability 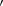 that 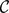 solves the given instance of CDH. First, we analyze the events needed for 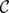 to succeed before the rewinding.

- *E*_1_: 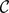 does not abort as a result of any of 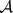
’s Extraction query.
- *E*_2_: 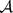 generates a valid and nontrivial aggregate signature forgery 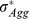 for 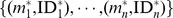.
- *E*_3_: Event *E*_2_ occurs and 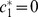, 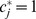 for 2≤ *j* ≤ *n*, where for each *i*, 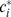 is the c-component of the tuple containing ID*_i_* on the 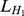.

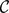
 succeeds before the rewinding if all of these events occur. The probability 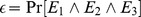 is decomposed as
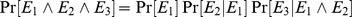



The following claims give a lower bound for each of these terms.

#### Claim 1

The probability that algorithm 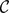 does not abort as a result of 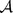
’s Extraction query is at least 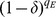. Hence we have 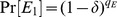.

#### Proof

Since 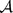 makes at most *q_E_* queries to the Extraction oracle and 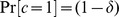, the probability that algorithm 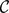 does not abort as a result of 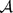
’s Extraction queries is at least 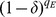.

#### Claim 2

If 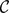 does not abort as a result of 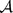
’s Extraction query, then 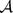
’s view is identical to its view in the real attack. Hence, 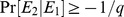.

#### Proof

Since the probability that 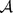 generates a valid and nontrivial signature for 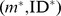 without asking *H*_2_ oracle in advance is less than 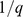, the probability that 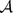 outputs a valid forgery 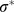 after querying 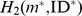 is at least 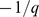.

#### Claim 3

The probability that 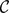 does not abort after 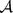 outputs a valid and nontrivial forgery is at least 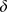. Hence, 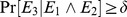.

#### Proof

After 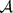 outputs a valid and nontrivial forgery, algorithm 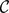 does not abort if and only if 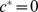. Since 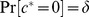, the probability that 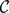 does not abort is at least 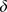.

Combining all of the above results, the probability 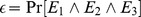 is at least


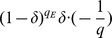


Therefore, in the first run of 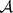, 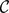 does not abort with probability.



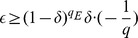



According to the general forking lemma [25], the probability that 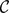 obtains two successful forgeries of 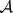 and does not abort is



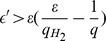
where 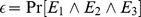. When 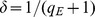, 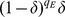 is maximized at
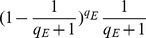


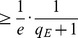
.

Therefore, the probability of solving the CDH problem is



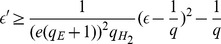
which is non-negligible if  is non-negligible.

Algorithm 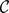
’s running time is roughly the same as 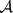
’s running time plus the time it takes to respond to hash queries, Extraction queries and Signature queries, and the time to transform 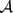
’s final forgery into the CDH solution. The *H*_1_ query requires a scalar multiplication. The Extraction query requires two scalar multiplications. The Signature query requires 4 scalar multiplications and the output phase requires a scalar multiplication and two inversions. Hence, the total running time is at most 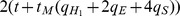 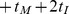.

## A New Identity-Based Aggregate Signature Scheme

### 5.1 Proposed identity-based aggregate signature scheme

Now, we construct an IBAS scheme using our basic IBS scheme constructed in the previous section.

- **Setup, Extract, Sign, Verify.** These algorithms are the same as those in our proposed IBS scheme.
- **Agg.** Begin with *n* signatures 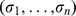 along with *n* message-identity pairs 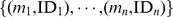 where 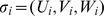 is the individual signature on message 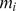 for identity 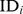,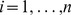. The aggregate signature generator computes 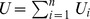, 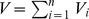 and 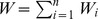, and outputs 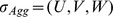 as an aggregate signature for message-identity pairs 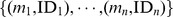.
- **AggVerify.** To verify the validity of an aggregate signature 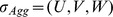 for message-identity pairs 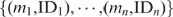, the verifier computes 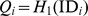, 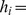 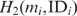, for 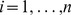, and checks.
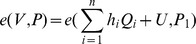

and
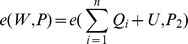



If both the equations hold, then the aggregate signature *σ_ Agg_* is valid.

### 5. 2 Security proof of the IBAS scheme

In this subsection, we are going to prove the security of our identity based aggregate signature scheme. The proof outline is as follows.

We assume on the contrary that our IBAS scheme is not EUF-IBAS-CMA secure. That is, assume there exists a polynomial time bounded adversary 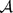 who can forge a signature in IBAS under the adaptive chosen message and chosen identity attacks. The proof’s goal is to show that under this assumption, our IBS scheme is not EUF-IBS-CMA secure.

#### Theorem 2

If there exists an adversary 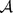 who has an advantage  in forging an aggregate signature of our IBAS scheme in the chosen aggregate modeled by Game II within a time at most *t*, after asking at most 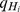 times *H_i_* (*i*  = 1, 2) queries, 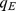 times Extraction queries, 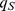 times Signature queries and at most *N* signers, then there exists an algorithm which in forging a signature of our IBS scheme in an attack modeled wins Game I within time.
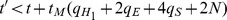
and with advantage



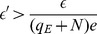
where *e* and 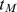 denote the same quantities as in Theorem 1.

#### Proof

Here we follow the idea from [17], [26], [27]. Suppose that 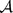 is a forger who breaks the IBAS scheme. By using 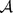, we will construct an algorithm 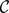 which outputs a forgery of our IBS scheme. Algorithm 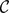 performs the following simulation by interacting with the adversary 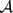.

#### Setup

It is the same as that described in the proof of Theorem 1.

#### *H*_1_ queries

To respond to *H*_1_ queries, 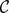 maintains a list 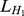 of tuples (ID, *t*, *c*, *Q*), which is initially empty. When 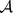 queries the oracle *H*_1_ on ID, 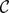 responds as follows:

1. If the query ID already appears on the 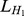 in a tuple (ID, *t*, *c*, *Q*), 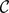 responds with *H*_1_(ID) = *Q*.
2. Otherwise, 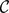 picks a random coin 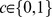 with Pr[*c* = 0]  =  *δ*.
  - If *c* = 0 then 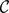 chooses 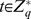 and computes *Q* = *t*(*bP*).
  - If *c* = 1 then 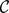 chooses 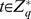 and computes *Q* = *tP*.

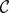
 adds the tuple (ID, *t*, *c*, *Q*) to the 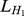 and responds to 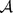 with *H*_1_(ID) = *Q*.

#### *H*_2_ queries, Extraction queries, Signature queries

When 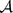 make *H*_2_ queries, Extraction queries, Signature queries, 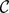 responds as those defined in the proof of Theorem 1.

#### Forgery

Eventually, 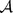 outputs an aggregate signature 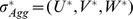 together with 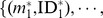 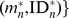.


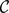
 recovers the corresponding tuples 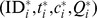 from the 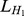 and the corresponding tuples (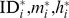) from the 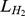 for all *i*, 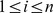.

It requires that there exists 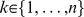 such that 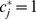 for *j* = 1, …, *n*, 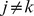, 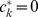 (without loss of generality, we let 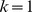), 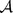 has not made a query Signature oracle on 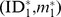 and 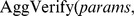 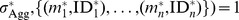. Therefore, the aggregate signature 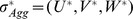 should satisfy the aggregate verification equations.
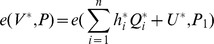


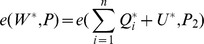




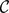
 sets 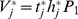 and 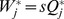. Obviously 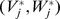 satisfy the equations 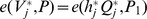 and 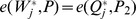 for 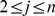. Then, 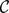 constructs  as 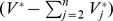 and 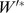 as 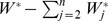. 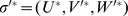 is a valid individual signature on 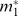 for 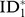 since it satisfies the verification equations as follows:
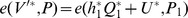


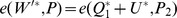



Finally, 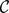 outputs 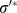 as a forgery of the IBS scheme.

#### Probability analysis

Similar to the analysis in **Theorem 1**, we analyze three events needed for 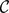 to succeed.

- *E*_1_: 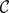 does not abort as a result of any of 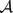
’s Extraction query.
- *E*_2_: 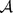 generates a valid and nontrivial aggregate signature forgery 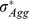 for 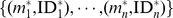.
- *E*_3_: Event *E*_2_ occurs and 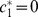, 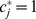 for 2≤ *j* ≤ *n*, where for each *i*, 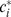 is the c-component of the tuple containing ID*_i_* on the 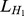.

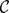
 succeeds if all of these events happen. The probability Pr[*E*_1_∧*E*_2_∧*E*_3_] is the same as in Theorem 1

#### Claim 1

The probability that 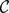 does not abort as a result of 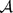
’s Extraction query is at least 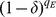. Hence, 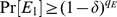.

#### Claim 2

If 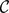 does not abort as a result of 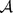
’s Extraction query and Signature queries, then 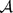
’s view is identical to its view in the real attack. Hence, 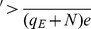.

#### Claim 3

The probability that 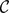 does not abort after 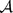 outputs a valid and nontrivial forgery is at least 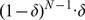.

#### Proof

Algorithm 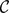 will abort unless 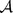 generates a forgery such that 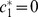 and 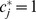 for 2≤ *j* ≤ *n*. Thus, 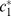 = 0 occurs with probability *δ*. And the probability that 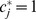, for 2≤ *j* ≤ *n*, is at least 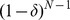. Therefore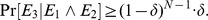.

Combining all of the above results, the advantage 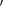 that 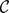 produces the correct answer is at least 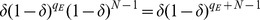 which is maximized at 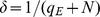. Therefore, the advantage 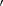 is


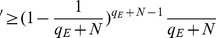




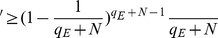
as required.

With Theorems 1 and 2, we can get the conclusion that the proposed IBAS scheme is secure against adaptively chosen-message and chosen-identity attacks under the hardness assumption of CDH problem in the random oracle model.

### 5.3 Performance analysis

Computation cost and aggregate signature size are two important parameters affecting the efficiency of an IBAS scheme. In this section, we compare our scheme with the existing unrestricted identity-based aggregate signature schemes [9], [14]–[17], [19], [20] from the aspects of aggregate signature size and computation cost in signature phase and aggregate signature verify phase, respectively. Detailed comparisons are summarized in Table 1. Here we only consider the costly operations (i.e., pairing operation, MapToPoint hash operation and multiplication operation in 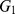) and omit the computational efforts which can be pre-computed. We use notations as follows:

**Table 1:** Comparisons of computation cost and aggregate signature size.

Scheme	Sign Time	AggVerify Time	Aggregate Signature Size
Cheon et al. [9]	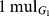	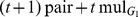	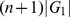
Xu et al. [14]	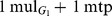	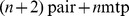	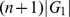
Herranz [15]	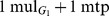	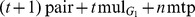	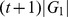
Kar [16]	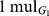	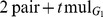	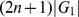
Shim [17]	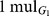	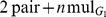	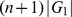
Kang [19]	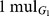	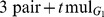	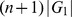
Hohenberger et al. [20]	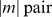	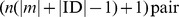	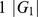
Our scheme	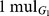	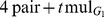	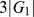

- 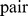: the time for performing a pairing operation
- 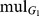: the time for performing a scalar multiplication in group 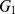.
- 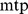: the time for performing a map-to-point hash operation
- 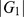: the length of element in group 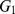.
- 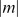: the length of the message *m.*
- 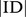: the length of the identity ID
*- t*: the number of distinct signers.
*- n*: the number of aggregated signatures.

From Table 1, we can see that the aggregate signature length of both of the scheme in [20] and our scheme is the same as that of a single individual signature regardless of the number *n* of signatures while that of the other schemes is directly proportional to either the number *n* of signatures or the number *t* of signers.

We also can observe that although the aggregate signature size overhead of Hohenberger et al.’s scheme [20] is better than that of ours (which is the shortest among the protocols under comparison), their scheme is less efficient in signing and aggregate verifying, which requires 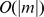 pairing operations to generate a signature and 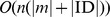 pairing operations to verify an aggregate signature. Our IBAS scheme requires no pairing operations for the signer and only four pairing operations for the verifier. As the pairing computation is the most time consuming in pairing-based cryptosystems [28], the computation overhead in our scheme is much faster than that in the scheme [20]. Therefore, the proposed scheme is more practical.

## Conclusions

In this paper, we proposed a new identity-based signature scheme that is provably secure in the random oracle model under the CDH assumption. We constructed an identity-based aggregate signature scheme using our IBS as the base signature scheme. The proposed IBAS enjoys significant advantages: aggregation is very general in that it allows for the aggregation of any multiple signatures from various users on various messages into a single compact signature; the aggregation operation does not require any restricted; AS meets the merit of signatures in ID-PKC which is free from the public key certificate management burden. The most important point is the compared with previous unrestricted IBAS schemes, our proposed scheme is the first IBAS scheme which satisfies both constant length aggregate signature and constant pairing operations. The security analysis has been provided and shown that the proposed schemes are secure against adaptive chosen-message attack and chosen-identity attack in the random oracle model. These features render our IBAS scheme an efficient solution to reduce bandwidth and storage, and are especially attractive for mobile devices like sensors, cell phones and PDAs where communication is more power-expensive than computation and contributes significantly to reducing battery life. Moreover, our scheme can adaptively work as a multi-signature scheme or a proxy signature scheme or a sequential aggregate scheme without any modifications.
